# Improved Fully 3D-Printed SIW-Based Sensor for Non-Invasive Glucose Measurement

**DOI:** 10.3390/s25082382

**Published:** 2025-04-09

**Authors:** Abdelhak Hamid Allah, Guy Ayissi Eyebe, Frédéric Domingue

**Affiliations:** Department of Electrical and Computer Engineering, Université du Québec à Trois-Rivières, Trois-Rivières, QC G9A 5H7, Canada

**Keywords:** SIW, 3D printing, microwave sensor, glucose monitoring, non-invasive, diabetes

## Abstract

This paper presents a fully 3D-printed microfluidic microwave sensor based on substrate-integrated waveguide (SIW) technology for detecting glucose levels in liquid, aimed at monitoring diabetic patients. The sensor’s design features a circular SIW cavity with an integrated sample holder placed in the cavity’s center, maximizing the electric field disturbance from the liquid under test (LUT). Operating in the *TM*_010_ mode at a resonance frequency around 5.740 GHz, the sensor detects glucose concentrations by measuring resonance frequency shifts in the *S*_11_ reflection response across glucose levels. A conductive sheath partially covers the sample holder to enhance sensitivity and improve the limit of detection (LOD) by increasing field penetration into the LUT. Fabricated using an additive manufacturing electronics (AMEs) method, the sensor is produced in a single pass without post-processing. The experimental validation confirms its high sensitivity of 1.218 MHz/(mg/dL) and a low limit of detection of 0.774 mg/dL in the glucose concentration range (10–200 mg/dL), reflecting typical Type 2 diabetes levels. The key advantages of the sensor include its compactness, enhanced sensitivity and limit of detection, innovative manufacturing, and cost-effectiveness, supporting its potential as a non-invasive glucose monitoring tool. This study establishes a proof of concept for the in vitro measurement of glucose, demonstrating the sensor’s ability to provide accurate and reliable results in a controlled environment.

## 1. Introduction

Diabetes is a chronic metabolic disorder characterized by elevated blood glucose levels, resulting from either insufficient insulin production by the pancreas or ineffective use of insulin by the body. Insulin plays a crucial role in regulating blood sugar levels. Without proper management, diabetes can lead to severe complications, such as damage to the eyes, heart, blood vessels, kidneys, and nerves [1]. Type 2 diabetes is the most prevalent form, affecting about 90% of individuals with diabetes, while Type 1 diabetes and gestational diabetes are less common. The World Health Organization (WHO) reports that diabetes affected 451 million people globally in 2017, with projections indicating a rise to 693 million by 2045 for individuals aged 18–99 [2]. In North America, 37 million people are currently living with diabetes, and it is expected that 5 million Canadians will be affected by 2025 [3,4].

Patients with diabetes are classified into three groups based on their blood glucose levels: hypoglycemia (≤70 mg/dL), normoglycemia (70–140 mg/dL), and hyperglycemia (≥140 mg/dL) [5]. Regular monitoring of blood glucose levels is recommended by the WHO to maintain target reference values and prevent complications [3]. Continuous glucose monitoring is a critical strategy for enhancing care and prevention for diabetic patients [6].

The glucometer, the most commonly used method for measuring glucose, requires a blood sample, which is then analyzed on a test strip. While effective, this method is invasive, painful, and uncomfortable. Additionally, it incurs high costs due to the need for disposable test strips, and each sample can only be measured once, limiting its practical applications [7].

Recent research has explored the use of microwave components to analyze glucose in blood and other fluids, with resonators showing great promise in detecting glucose levels. These resonators work by sensing changes in the electric field distribution and resonance characteristics when dielectric samples, like glucose in liquids, are introduced into the resonator’s sensing area. Various resonator-based methods, including antennas [8,9,10,11], dielectric resonator antennas [12,13], waveguides [14,15], split-ring resonators (SRRs) [16,17,18], and substrate-integrated waveguides (SIWs) [19,20,21,22], have all demonstrated effective glucose detection in aqueous solutions and blood.

In resonator-based glucose sensors, two primary measurement methods are commonly used: the immersive type, where the resonator is directly immersed in the glucose LUT [23,24], and the sample holder type, where the LUT is placed in a holder within the resonator’s sensitive area [17,19,25,26]. While the immersive method allows for direct contact with the LUT, it may introduce contamination risks, whereas the sample holder method avoids this but often limits the electric field distribution to a two-dimensional cross-section. This restriction reduces both sensitivity and efficiency, as it prevents the sensor from ensuring full contact with the LUT, which is crucial for accurate glucose measurements.

To address this limitation, this paper proposes a novel resonator-based sensor that utilizes the sample holder type. The key innovation involves adding a conductive sheath around the sample holder. This sheath enhances the electric field distribution in three dimensions, enabling the electric field to penetrate the LUT more effectively, acting as a waveguide section. As a result, the sensor’s sensitivity is increased, the limit of detection is reduced, and the measurement accuracy is improved. Moreover, the sensor is fabricated using 3D additive manufacturing electronics (AMEs), a technique that allows for a fully integrated 3D fabrication process. This approach eliminates the need for post-manufacturing steps required in traditional methods, while also maintaining a compact and cost-effective design. By improving the electric field distribution through the conductive sheath and optimizing the manufacturing process with 3D printing, this approach leads to significant advancements in glucose sensor performance, and offers greater scalability for non-invasive monitoring.

The paper is structured as follows: Section 2 discusses the design of standard and optimized SIW-based sensors, detailing their operating principles, materials, and fabrication process using the AMEs technique. Section 3 outlines the experimental setup, sample preparation, and testing process. Section 4 presents and analyzes the results and effectiveness of the glucose monitoring approach. Finally, Section 5 concludes the paper.

## 2. SIW-Based Sensor Design and Fabrication

### 2.1. SIW-Based Sensor Design and Sensing Principle

The standard topology of the SIW-based sensor is characterized by a cylindrical resonant SIW cavity that includes a sample holder positioned at the center of the cavity. The sample holder has a volume of $V_{\mathrm{holder}}=\pi {\left(D_{\mathrm{hold}\_\mathrm{in}}/2\right)}^{2}H_{\mathrm{hold}\;}$. For measurement purposes, the cavity is fed by a microstrip line with dimensions of ($W_{\mathrm{feed}}\times L_{\mathrm{feed}}$). The operating principle of the proposed sensor is straightforward and involves the resonance of the cavity structure in the fundamental mode of the dominant (${\mathrm{TM}}_{010\;}$), which can be calculated by the following [27]:

(1) $$f_{r}=\frac{\mu_{mn\;}c}{2\pi r\sqrt{{\varepsilon_{r\;}\mu }_{r\;}}}$$

The root of the Bessel function, $\mu_{mn\;}$, along with the SIW cavity radius, r, and the permittivity and permeability of the dielectric material, $\varepsilon_{r\;}$ and $\mu_{r\;}$, respectively, play a crucial role in cavity’s resonant frequency. At the resonant frequency, the concentration of the electric field is highly localized at the cavity’s center, rendering this region highly susceptible to sample holder build-up. However, the presence of a loaded LUT alters the dielectric properties of the sensitive area, leading to a shift in the resonant frequency. The resulting variation can be expressed using the following equation [28].

(2) $$\frac{∆f_{r}}{f_{r}}=\frac{\int_{v_{c}}\;\left(\Delta \varepsilon \left|E_{1}\right|\left|E_{0}\right|+\Delta \mu \left|H_{1}\right|\left|H_{0}\right|\right)dv}{\int_{v_{c}}\;\left(\varepsilon_{0}{\left|E_{0}\right|}^{2}+\mu_{0}{\left|H_{0}\right|}^{2}\right)dv}$$

In (2), the cavity volume is denoted as $v_{c}$, and $∆f_{r}$, Δε, and Δμ represent the variation in resonance frequency, complex permittivity, and complex permeability, respectively. The permittivity and permeability of free space are denoted by $\varepsilon_{0}$ and $\mu_{0}$, respectively. The subscripts $E_{0}$, $H_{0}$, and $E_{1}$, $H_{1}$ refer to the electric and magnetic fields of the empty and loaded cavities, respectively. When a relatively small LUT is used, the electric and magnetic fields inside the resonator are considered unchanged before and after LUT loading. For dielectric materials, Δμ can be considered zero. Additionally, at the resonant frequency, the energy accumulated by the electric and magnetic fields in the resonant structure must be equivalent. Equation (2) is thus simplified under these circumstances [29]:

(3) $$\frac{∆f_{r}}{f_{r}}=\frac{\int_{v_{s}}\;\left(\Delta \varepsilon \left|E_{1}\right|\left|E_{0}\right|\right)dv}{2\int_{v_{c}}\;\left(\varepsilon_{0}{\left|E_{0}\right|}^{2}\right)dv}$$

$v_{s}$ represents the LUT sample volume. It is shown by Equation (3) that the resonant frequency shift is mainly determined by the electric field variation caused by the loaded LUT.

### 2.2. Sample Holder Performance Analysis

The performance of the sensor designed to detect a loaded LUT is highly influenced by the sensor’s geometry, particularly the size of the sample holder and the relative permittivity of the LUT. To enhance the sensor’s sensitivity while reducing its overall volume and cavity size, a detailed numerical examination was conducted. The analysis focused on evaluating the effect of the sample holder dimensions and the LUT’s relative permittivity on the sensor’s performance. This study employed full-wave 3D electromagnetic simulations using Ansys HFSS (High-Frequency Structure Simulator) (Ansys, Canonsburg, PA, USA) to model and optimize the sensor’s design.

The sensor performance was quantified by calculating the relative frequency shift, which represents the ratio of the resonance frequency of the LUT-loaded sensor to the empty sensor resonance frequency. This approach provided a clear indication of the interaction between the LUT and the electric field within the sensor structure.

**Figure 1:**
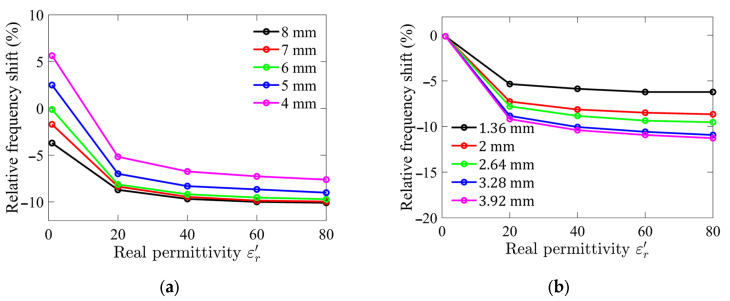
Simulated relative frequency shifts for different (**a**) sample holder diameters; and (**b**) sample holder heights as functions of relative permittivity of LUT.

Numerical simulation results are presented in Figure 1. The analysis revealed that as the diameter and height of the sample holder increase, the interaction between the LUT and the electric field intensifies, leading to a higher sensitivity. Specifically, the relative frequency shift remains constant once the sample holder diameter exceeds 6 mm, indicating that the LUT is effectively interacting with nearly all of the transverse electric fields. However, further increasing the sample holder diameter beyond this point results in diminishing returns, with the excess holder size having little effect on sensitivity and leading to inefficient use of the LUT.

The range of relative permittivity of interest in this study is between 40 and 80, corresponding to the typical permittivity of aqueous glucose solutions. Since the dielectric constant of water, the primary solvent, is approximately 78.5 at room temperature, and the addition of glucose slightly reduces this value depending on its concentration, this range was selected to evaluate the sensor’s performance under realistic conditions. Although the simulation was performed over a wider range of dielectric constants, from 1 to 80, to capture the overall trends and identify any unexpected behavior.

Based on these findings, the optimized dimensions of the sample holder were determined to be a diameter of 6 mm and a height of 2.64 mm. These optimized parameters resulted in a sample holder volume of approximately 74 µL, representing a compact yet effective design for the sensor.

### 2.3. Optimized SIW-Based Sensor Design

**Figure 2:**
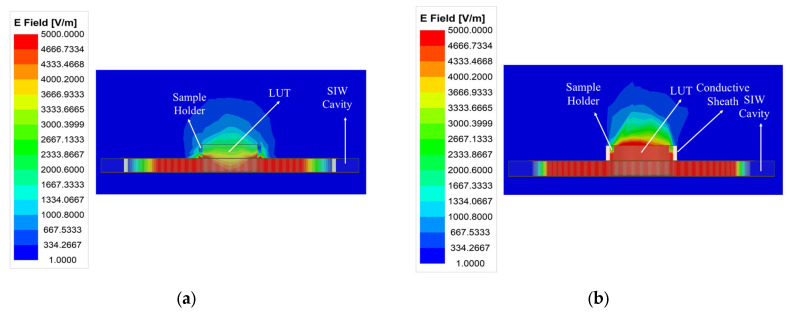
A comparison of the electric field amplitude distribution at the resonant frequency of (**a**) the standard SIW-based sensor and (**b**) the optimized SIW-based sensor.

**Figure 3:**
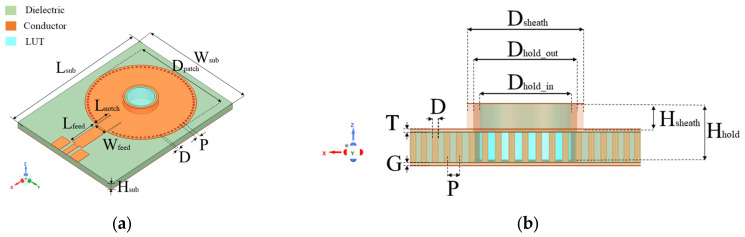
(**a**) 3D view; and (**b**) sectional view of proposed SIW-based sensor. Geometric parameters in (mm) are as follows: *L_sub_* = 43.56, *W_sub_* = 33, *H_sub_* = 1.5, *L_feed_* = 9, *W_feed_* = 3.5, *L_notch_* = 0.3, *P* = 1.04, *D* = 0.5, *D_hold_in_* = 6, *D_hold_out_* = 7, *H_hold_* = 2.64, *D_sheath_* = 8, *H_sheath_* = 1.47, and *T* = *G* = 0.03.

The weak electric field distribution within the microfluidic sample holder limits the performance of the standard SIW-based topology sensor, leading to a degradation in detection sensitivity and detection limit. As illustrated in Figure 2, the distribution and penetration of the electric field into the LUT can be enhanced by adding a conductive sheath around the sample holder. This modification improves the interaction between the LUT and the electric field, resulting in increased sensitivity, which is considered the most critical performance factor for microfluidic sensors, consequently enhancing the limit of detection. Furthermore, the conductive sheath acts as a waveguide section placed over the SIW cavity, confining the electric field within the holder and preventing stray radiation. To achieve this, a conductive sheath was integrated into the optimized SIW-based sensor, as shown in Figure 3.

### 2.4. Materials and Additive Manufacturing Electronic Method

**Figure 4:**
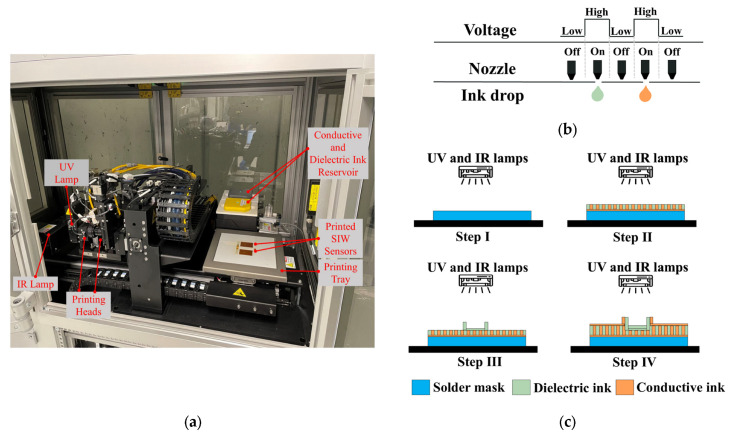
(**a**) One-step additive manufacturing system; (**b**) operating principle of piezoelectric inkjet; and (**c**) printing process used to manufacture proposed SIW-based sensor.

The DragonFly™ LMD printer (Nano Dimension Ltd., Ness Ziona, Israel) [30] is utilized for fabricating the proposed SIW-based sensor. As illustrated in Figure 4a, the printer has dimensions of (x = 1400 × y = 800 × z = 1800) and features two printheads for conductive and dielectric printing. The conductive print head is linked to a reservoir of silver nanoparticle-based ink with a conductivity of (11.2 × 10^6^ S/m at 20 °C) (Nano Dimension Ltd., Ness Ziona, Israel), which is utilized for conductive printing. On the other hand, the dielectric print head is linked to a reservoir of ultraviolet (UV) curable acrylic ink (Nano Dimension Ltd., Ness Ziona, Israel) with dielectric properties (dielectric constant $\varepsilon_{r}$ = 2.77, and tangential loss tanδ = 0.012 at 5.740 GHz), used for dielectric printing.

The AMEs technology employed by the printer is the piezoelectric inkjet, which utilizes liquid ink deposition and 512 nozzles per printhead. As Figure 4b demonstrates, an electrical voltage is supplied to the liquid to generate a pressure pulse, which ejects a 4 pL droplet of conductive or dielectric ink out of the nozzle. The printing tray can accommodate a 3D design with a maximum size of (x = 160 × y = 160 × z = 3) mm^3^, with a minimum resolution thickness of 0.3 µm and 2.6 µm for the conductive and dielectric layers, respectively.

The printing process for the one-step additive manufacturing system, which involves the creation of conductive and dielectric materials, is depicted in Figure 4c. Initially, a thin solder mask is deposited on the bottom of the structure. Subsequently, predetermined patterns are followed to deposit the dielectric and conductive inks directly onto the solder mask to produce the structure’s layers. Finally, infrared (IR) and ultraviolet (UV) lamps are activated to solidify the conductive and dielectric inks.

## 3. Experimental Measurements

### 3.1. Experimental Setup

**Figure 5:**
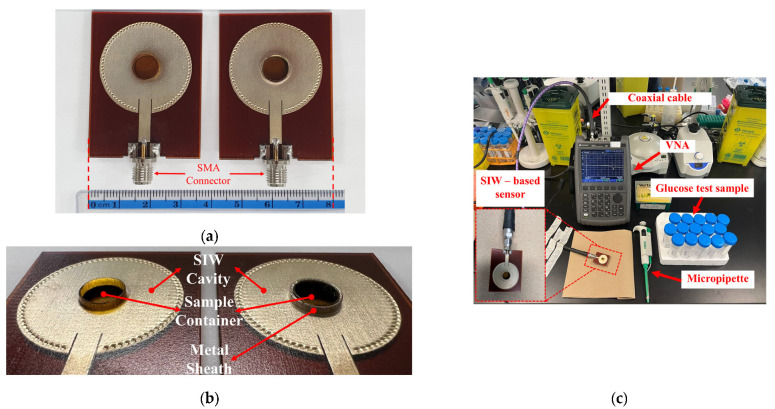
(**a**) Fabricated standard SIW-based sensor (right) and optimized SIW-based sensor (left); (**b**) zoom-in of sensors; (**c**) Measurement setup for sensors’ test.

To conduct a performance analysis of the 3D-printed standard and the optimized SIW-based sensors, as illustrated in Figure 5a,b, two 50-Ω subminiature-A (SMA) version connectors were affixed to the sensors’ supply line for experimental purposes. Subsequently, a stable experimental setup was established, as depicted in Figure 5c. The sensors were connected via a coaxial cable to a Keysight Technologies N9928A vector network analyzer (VNA) (Keysight Technologies, Santa Rosa, CA, USA) for data collection. The VNA was calibrated using the Agilent 85521A (Agilent Technologies, Santa Clara, CA, USA) module’s standard Short-Open-Load (SOL) calibration approach to ensure reliable and repeatable measurements. The reflection scattering responses (*S*_11_ coefficient) were recorded by sweeping the frequency at 1001 points within the 4–7 GHz frequency range. All measurements were conducted in a biomedical laboratory maintained at an ambient, climate-controlled room temperature of 25 ± 1 °C—widely recognized in the literature as the standard condition for microwave sensor testing. To ensure accurate and consistent measurements by minimizing the effects of temperature variations on the dielectric properties of the samples, the laboratory is equipped with a dedicated HVAC (Heating, Ventilation, and Air Conditioning) system. This system features a precision thermostat and a feedback-controlled heating and cooling mechanism, enabling fine regulation of room temperature throughout the entire measurement process.

### 3.2. Experimental Empty Measurement

**Figure 6:**
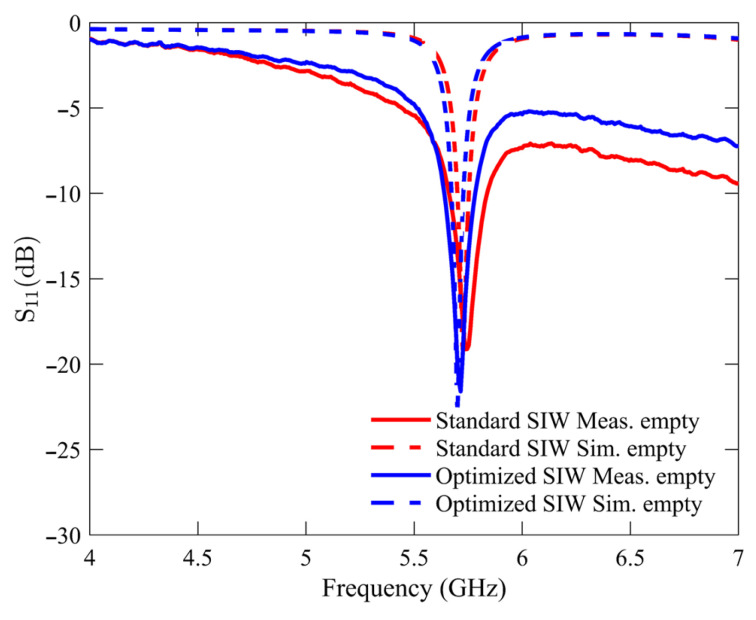
Simulated and measured reflection scattering responses (*S*_11_ coefficient) for standard and optimized SIW-based sensors in empty sample holder.

The initial experimental measurement was performed under an unloaded condition with an empty sample holder. This particular condition was utilized as the reference point for all subsequent measurements of the glucose samples. Figure 6 compares the *S*_11_ response of the standard SIW-based sensor and the optimized SIW-based sensor. The plotted data indicates that both sensors exhibit unloaded frequency resonances at approximately 5.740 GHz, with the standard SIW-based sensor displaying a peak resonance depth of −19.12 dB and the optimized SIW-based sensor demonstrating a step depth of −21.6 dB. The measurements and numerical simulations exhibit a high level of agreement, with minimal variations attributable to manufacturing and soldering tolerances.

### 3.3. Experimental Glucose Monitoring Measurements

The proposed sensing approach for measuring glucose concentrations in aqueous solutions was validated by performing the second experimental measurement step in a loaded sample holder, in which different glucose–water samples were injected. These samples were prepared by mixing D-(+) glucose powder and deionized (DI) water and were utilized to simulate the actual blood glucose concentration of Type 2 diabetes. This approach can be justified since water constitutes approximately 50% of the total blood volume and contains various amounts of other crucial components, such as glucose, Ca, Na, Cl, and K, which significantly contribute to the dielectric characteristics of blood through their ionic conductivity and polarizability [31]. The glucose–water samples, comprising 14 different samples covering the actual blood glucose concentration levels for Type 2 diabetes, namely the following: hypoglycemia: *C_gl,_*_2_ < 70 mg/dL, normoglycemia: 70 mg/dL ≤ *C_gl,_*_2_ < 140 mg/dL, and hyperglycemia: 140 mg/dL ≤ *C_gl,_*_2_, were prepared in the laboratory using a micropipette and the dilution formula $C_{\mathrm{gl},1}V_{\mathrm{gl},1}=C_{\mathrm{gl},2}V_{\mathrm{gl},2}$, where the volume *V_gl,_*_1_ was obtained from a glucose stock solution with a concentration of *C_gl,_*_1_ = 1000 mg/dL, and the volume *C_gl,_*_2_ = 50 mL was calculated with the desired glucose concentrations varying from *C_gl,_*_2_ = 10 to 200 mg/dL.

The glucose measurements were performed by filling the sample holder with a sample volume of 74 µL using a micro pipetting procedure. The corresponding *S*_11_ was recorded after the micro pipetting was immediately stopped (<6 s). Distilled water was used to rinse the sample holder after each measurement step, and laboratory tissue was used to clean it and remove the previously tested sample, ensuring a fair comparison of resonance response before loading the following sample.

## 4. Results and Discussion

### 4.1. Experimental Glucose Monitoring Results and Sensing Model

**Figure 7:**
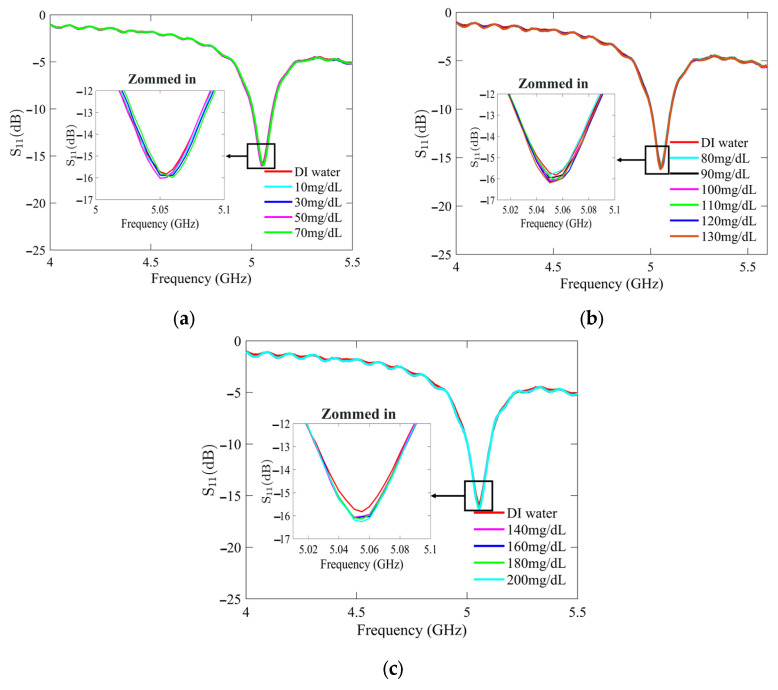
Measured reflection scattering responses (*S*_11_ coefficient) of standard SIW-based sensor for tested glucose concentrations: (**a**) hypoglycemia; (**b**) normoglycemia; and (**c**) hyperglycemia.

**Figure 8:**
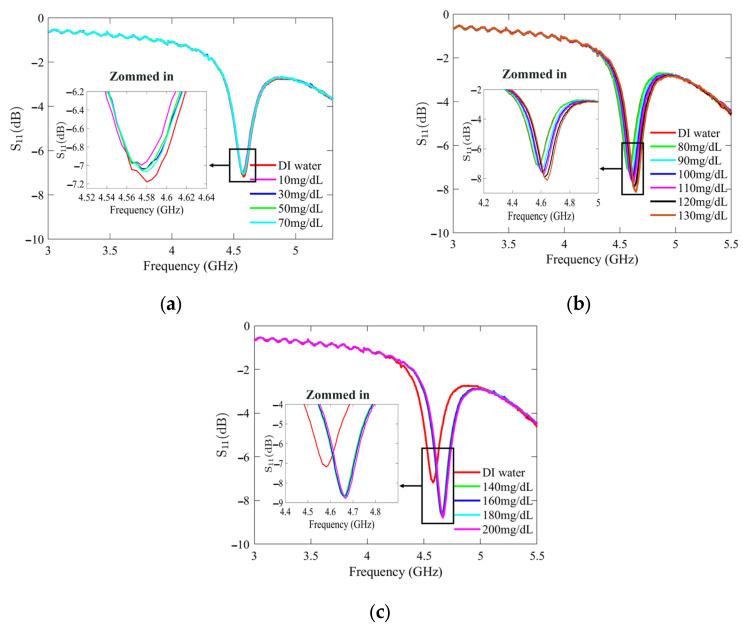
Measured reflection scattering responses (*S*_11_ coefficient) of optimized SIW-based sensor for tested glucose concentrations: (**a**) hypoglycemia; (**b**) normoglycemia; and (**c**) hyperglycemia.

Figure 7 and Figure 8 present the recorded changes in the *S*_11_ for the standard and optimized SIW-based sensors, respectively. It is worth noting that with an increase in the glucose concentration, the resonance frequencies of *S*_11_ move towards higher frequencies while exhibiting a visible change in amplitude. This outcome is predictable as the permittivity properties of the glucose sample induce slight changes in the overall permittivity of the sample holder. Accordingly, the shift in the resonant frequency is regarded as the principal detection parameter. The frequency difference between the DI water and different glucose concentrations is very small, primarily due to the slight dielectric permittivity variation between the DI water and glucose solutions.

**Figure 9:**
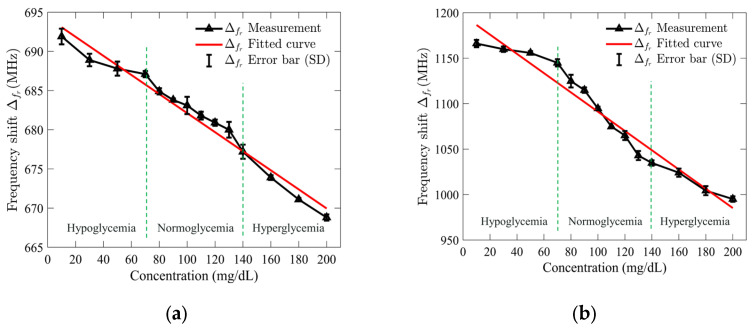
Measured resonance frequency shift of (**a**) standard SIW-based sensor; and (**b**) optimized SIW-based sensor versus tested glucose concentrations.

Once the *S*_11_ response results were recorded, the collected data were used to calculate the frequency shifts by subtracting the resonance frequency of the empty sensor from that of each glucose concentration, which were then plotted to assess the relationship. A regression analysis was performed to evaluate the relationship between the shift in resonance frequency and the concentrations of the tested glucose solutions. Figure 9a,b illustrates the outcomes of the applied analysis for the standard and optimized SIW-based sensors. The analysis revealed a linear relationship between the resonance frequency shift and glucose concentrations, with high correlation coefficients (R^2^) of 0.97 and 0.95 for the standard and optimized SIW-based sensors, respectively.

To demonstrate both repeatability and selectivity, the *S*_11_ response of the sensors was recorded five times under consistent test conditions, and the standard deviation (SD) error bar was applied to quantify the variability of the measured resonance frequency shift. For the standard SIW-based sensor, the maximum error was ±1 MHz at a glucose concentration of 130 mg/dL, while the optimized sensor showed a maximum error of ±7 MHz at 80 mg/dL. These results suggest that the standard sensor is more precise at higher concentrations, while the optimized sensor performs well at lower concentrations with slightly more variability. The small error values indicate minimal test error, confirming the sensor’s high repeatability and stability under consistent conditions. The ability to detect small frequency shifts, especially at higher glucose concentrations, highlights the sensor’s excellent selectivity, making it a reliable and sensitive tool for glucose monitoring.

In order to develop the sensing mathematical model of the proposed sensor, the regression set data shown in Figure 9 was utilized. The mathematical model for the standard SIW-based sensor was obtained by using the frequency shift, which is as follows:

(4) $$C_{gl}\left(\mathrm{mg}/\mathrm{dL}\right)=5.4669\;\times \;10^{3}-\;7.8740\;\times \;∆f_{r}\left(\mathrm{MHz}\right)$$

The sensing model obtained for the optimized SIW-based sensor is given by the following:

(5) $$C_{gl}(\mathrm{mg}/\mathrm{dL})=1.1292\;\times \;10^{3}-0.9434\;\times \;∆f_{r}\left(\mathrm{MHz}\right)$$

### 4.2. Sensor Glucose Monitoring Reliability Validation

In order to investigate the reliability of the sensors in glucose monitoring applications, the measured and actual glucose concentrations were plotted using the Clarke error grid. This grid was developed in 1987 by biologist Clarke to assess the reliability of commercial glucometers [32]. On the grid, the X-axis represents actual glucose concentrations, while the Y-axis represents measured glucose concentrations. The glucose concentration errors are divided into five zones: A, B, C, D, and E. Zone A, below 20%, is considered optimal for glucose concentrations. In contrast, zone B, above 20%, is not compliant. Finally, zones C, D, and E are considered unsafe for glucose concentrations and may harm the patient’s health.

**Figure 10:**
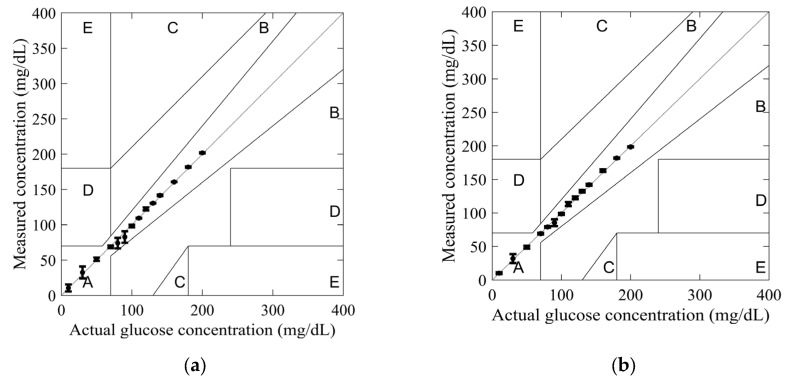
Clarke’s error grid obtained from measurements for tested glucose concentrations: (**a**) standard SIW-based sensor; and (**b**) optimized SIW-based sensor.

The glucose concentration values obtained from models (4) and (5) were used to plot the Clarke error grid in Figure 10. All measured values for both SIW-based sensors fell within Zone A, confirming their reliability for glucose monitoring applications.

The highest measurement uncertainty was observed at a glucose concentration of 90 mg/dL using model (4), with an uncertainty of 8.19%. In comparison, at 30 mg/dL, model (5) exhibited a lower uncertainty of 6.66%. Across most tested glucose concentrations, model (5) demonstrated greater accuracy, highlighting the effectiveness of the added conductive sheath. This improvement is further substantiated by the observed variations in the *S*_11_ amplitude and resonant frequency, as depicted in Figure 8. By integrating resonant frequency shifts and reflection responses into a mathematical model, measurement accuracy can be further enhanced.

### 4.3. Sensor Performance Comparisons

To demonstrate the effectiveness of our proposed SIW-based sensor, Table 1 presents a comparative analysis with existing literature on several crucial sensing factors. Our sensor outperforms others in detecting slight variations in glucose concentrations within the Type 2 diabetes range, achieving a high detection sensitivity and a low detection limit. Specifically, the sensitivity improves from 0.122 MHz/(mg/dL) to 1.218 MHz/(mg/dL), while the detection limit decreases from 7.721 mg/dL to 0.774 mg/dL when comparing the standard SIW-based sensor to the optimized SIW-based sensor. The sensitivity ($S=∆f_{r}/∆c_{\mathrm{gl}}$) [33] is measured by the change in resonant frequency per unit alteration in the tested glucose concentration. The limit of detection represents the smallest detectable concentration of the target substance in a sample and is determined using the formula (LOD=k.σ/S) [33], where k is a constant related to the confidence level (typically set to 3), σ represents the standard deviation of the blank, and S is the sensitivity.

Beyond its high sensitivity and low detection limit, our sensor offers several notable advantages, making it highly suitable for real-time blood glucose monitoring. These advantages include non-destructiveness, affordability, a novel sensitivity enhancement technique, and the ease of fabrication through advanced 3D printing technology. The integration of the entire sensor system, including the SIW cavity, sample holder, and conductive sheath, is achieved in a single manufacturing step. This approach eliminates the need for multiple equipment manipulations and post-processing steps, which are typically required in traditional fabrication methods, such as PCB manufacturing. By simplifying the fabrication process, our method reduces production complexity, minimizes the risk of human error, and ensures consistent sensor performance. Additionally, the use of 3D printing enhances design flexibility, allowing for easy modifications and rapid prototyping to adapt the sensor for various applications.

Although the current study establishes a proof of concept for the detection of in vitro glucose for Type 2 diabetes, demonstrating the sensor’s potential for accurate and reliable measurements, several avenues remain open for future exploration to further enhance sensor performance. Extending the testing to include more duplicates can help validate the stability of the sensor and quantify measurement errors over a wider range of samples, while testing with real blood samples will assess the impact of other blood constituents and variations in density on sensor accuracy. Additionally, minimizing the liquid volume required for testing could be achieved by miniaturizing the SIW cavity through the introduction of strategically placed slots on the upper cavity surface, which would concentrate the electric field in a more confined region, thereby increasing the sensitivity and improving detection limits. Further investigations could also explore alternative materials and optimize geometric designs to enhance the sensor’s robustness and accuracy under varying environmental conditions. These improvements would contribute to the development of a more reliable, precise, and adaptable sensor for real-time glucose monitoring.

## 5. Conclusions

This paper introduces a microfluidic microwave SIW-based sensor for monitoring liquid glucose with enhanced sensitivity. The detection structure consists of conductive side vias, a pair of top and bottom conductive plates, and a sample holder integrated into the SIW cavity-sensitive area to detect glucose. A conductive sheath, partially covering the sample holder, is included to improve the electrical field interrogation and detection sensitivity when loaded with the LUT. The resonant frequency of the reflection responses (coefficient *S*_11_) changes with the presence of the LUT, which determines the sensing principle. The fabricated sensor’s sensing principle and performance were tested using experimental laboratory measurements on different liquid samples simulating synthetic blood with low glucose concentrations (10–200 mg/dL), corresponding to Type 2 diabetes. The proposed sensor exhibits a high sensitivity of approximately 1.218 MHz/(mg/dL) and a low detection limit of 0.774 mg/dL. With its simple design, compact size, and innovative sensitivity enhancement method, this sensor holds significant potential for advancing high-sensitivity in vitro sensors for body fluids and non-invasive blood glucose monitoring in diabetes management.

## Figures and Tables

**Table 1:** Comparison between state-of-art microwave sensors used for glucose monitoring.

Ref.	Sample V (µL)	*C_gl_* (mg/dL)	Oper. Freq. *f_r_* (GHz)	Sensing Parameter	S (MHz per (mg/dL) or dB per (mg/dL))	LOD (mg/dL)	Fabrica. Technology
[33]	N/A	50–500	3.4–4.2	*f_r_* (*S*_11_)	2.45 × 10^−1^	7.7	PCB
[34]	N/A	100–120	1.17	*f_r_* (*S*_21_)	5.4 × 10^−2^	10	PCB
[35]	N/A	0–80	2.56	*f_r_* (*S*_21_)	2.00 × 10^−2^	50	PCB
[36]	N/A	1800–18,000	1.156	*S*_21_	1.33 × 10^−5^	18	PCB
[36]	500	0–250	6	*S*_21_	3.7 × 10^−3^	1.92	PCB
[37]	N/A	30–50	1.5	*S*_11_	4.9 × 10^−1^	35	PCB
[38]	7500	78–5000	1.4–1.9	*S*_11_	1.800 × 10^−3^	N/A	PCB
[39]	125	100–1000	4.8	*f_r_* (*S*_11_)	1.400 × 10^−2^	N/A	PCB
[40]	500	20–120/100–600	7.8	*S*_11_	1.200 × 10^−2^	N/A	PCB
[41]	600	40–140	2.45	*f_r_* (*S*_21_)	(4.5–9.5) × 10^−1^	1.00	PCB
[17]	500	400–1200	7.8	*f_r_* (*S*_11_)	(2.74–3.34) × 10^−1^	N/A	PCB
[42]	N/A	105–225	9.20	*f_r_* (*S*_21_)	0.18 × 10^−1^	8.01	PCB
[43]	40	0–400	4.4	*f_r_* (*S*_21_)	13.00 × 10^−1^	N/A	PCB
[44]	N/A	250–16,000	2.4–2.5	*f_r_* (*S*_11_)	7.8285 × 10^−3^	N/A	PCB
[45]	1500	50–350	22–32	*f_r_* (*S*_12_/*S*_21_)	7.000 × 10^−2^	N/A	PCB
[22]	101	10–200	3.980	*S*_11_	4.17 × 10^−2^	7.194	AMEs 3D
T.W	74	10–200	5.715/ 5.745	*f_r_* (*S*_11_)	1.220 × 10^−1^/ 12.180 × 10^−1^	7.721/ 0.774	AMEs 3D

## Data Availability

Data are unavailable due to privacy restrictions.
